# New Zealand Hospital Board's Conference

**Published:** 1920-07-31

**Authors:** 


### New Zealand Hospital Board's Conference.

### Shall the State Establish a Hospital Service?

On June 1 and 2, 1920, there was held in Wellington, New Zealand, a Conference of representatives appointed from some thirty-nine Hospital and Charitable Aid Boards comprising all the hospital districts in the Dominion. The officers of the Department of Hospitals, Charitable Aid, and Public Health were present, and did excellent service in clearing away many misunderstandings in regard to problems in connection with the question of national health.

### Minister's Views of Bureaucratic Control.

The question of nationalisation of public hospitals was referred to by the Minister of Public Health in the course of his opening address. The Minister stated he was keen always for State undertaking, provided that the State was able to carry on better than private enterprise; but in connection with nationalisation of hospitals, or anything else, there are evils under a bureaucratic control as well as in other systems. Unless the State is prepared to pay for "brains" some £2,500 instead of £800, £900 or a £1,000 per annum, it could not hope for success. He is not prepared to undertake nationalisation without making reforms as to central management. Medical men, like school teachers, are not always business men, and in this fact will lie one weakness of the State trying to run the hospital service. He advocates separating the business aspect from the professional side, and unless the State is prepared to do this he would not counsel the extension of State enterprise relating to the hospitals. Owing to the demands on the Treasury being unprecedentedly large the Minister of Finance would hardly favour the proposal for the additional burden of cost of running the hospitals. The Medical Association has given the Dominion a lead, and he is of opinion that in the interests of public health, and the public generally, some modified form of national medical service should be instituted. Any system adopted should pay special attention to the needs of sparsely settled districts, mining communities, and poor people in the cities. There could be no question that this reform will come. Finance is the great difficulty, and the tremendous increases in the annual charges on the Treasury during the last five years prevents any reform being taken this year.

During the Conference a resolution on the following terms was introduced: "That, owing to the increasing demands by the public and sections of the public for further extension of hospital and nursing facilities, it is desirable that the State should establish a hospital service, so as to secure some uniformity as regards salaries and conditions of service for medical officers and nurses." The Inspector-General of Hospitals stated that a State Medical Service, and a very efficient one, could be instituted without nationalising the hospitals. It was pointed out by one delegate that there is an increasing tendency to lean more and more on the Department, which indicates that the people are travelling along the lines of nationalisation. It was resolved finally to refer the whole of the proposals to the Public Health Department for consideration, and to report to the various Hospital Boards throughout the Dominion.

